# Increases in theta CSD power and coherence during a calibrated stop-signal task: implications for goal-conflict processing and the Behavioural Inhibition System

**DOI:** 10.1017/pen.2019.10

**Published:** 2019-10-25

**Authors:** Thomas S. Lockhart, Roger A. Moore, Kim A. Bard, Lorenzo D. Stafford

**Affiliations:** Department of Psychology, University of Portsmouth, Portsmouth, UK

**Keywords:** conflict, BIS, theta, coherence, neuroticism

## Abstract

Psychologists have identified multiple different forms of conflict, such as information processing conflict and goal conflict. As such, there is a need to examine the similarities and differences in neurology between each form of conflict. To address this, we conducted a comprehensive electroencephalogram (EEG) analysis of Shadli, Glue, McIntosh, and McNaughton’s calibrated stop-signal task (SST) goal-conflict task. Specifically, we examined changes in scalp-wide current source density (CSD) power and coherence across a wide range of frequency bands during the calibrated SST (*n* = 34). We assessed differences in EEG between the high and low goal-conflict conditions using hierarchical analyses of variance (ANOVAs). We also related goal-conflict EEG to trait anxiety, neuroticism, Behavioural Inhibition System (BIS)-anxiety and revised BIS (rBIS) using regression analyses. We found that changes in CSD power during goal conflict were limited to increased midfrontocentral theta. Conversely, coherence increased across 23 scalp-wide theta region pairs and one frontal delta region pair. Finally, scalp-wide theta significantly predicted trait neuroticism but not trait anxiety, BIS-anxiety or rBIS. We conclude that goal conflict involves increased midfrontocentral CSD theta power and scalp-wide theta-dominated coherence. Therefore, compared with information processing conflict, goal conflict displays a similar EEG power profile of midfrontocentral theta but a much wider coherence profile. Furthermore, the increases in theta during goal conflict are the characteristic of BIS-driven activity. Therefore, future research should confirm whether these goal-conflict effects are driven by the BIS by examining whether the effects are attenuated by anxiolytic drugs. Overall, we have identified a unique network of goal-conflict EEG during the calibrated SST.

Conflict has become an area of increasing interest for psychologists and neuroscientists. This is perhaps due to the relevance of conflict to both functional cognition (e.g., decision making) and dysfunctional cognition (e.g., anxiety disorders) (see Cavanagh & Shackman, 2015; Gray & McNaughton, 2000). However, there is no one single accepted definition of conflict (e.g., Botvinick, 2007; Cavanagh & Frank, 2014; Cohen, 2014; McNaughton, DeYoung, & Corr, 2016). As such, there is a need to examine the neurology of each form of conflict individually to identify any differences and similarities.

The neurology of one form of conflict, information processing conflict (IPC), is already well understood. IPC can occur when an individual is deciding how best to respond, based on the available information. Examinations of IPC most commonly use response conflict tasks, such as the Stroop, Flanker and Simon tasks (Botvinick, Braver, Barch, Carter, & Cohen, 2001). During these tasks, each participant is given conflicting information about which response is correct, creating IPC (for a detailed explanation of each task, see Stins, Polderman, Boomsma, & de Geus, 2005). In his review, Cohen (2014) described two major patterns of electroencephalogram (EEG) activity during these response conflict tasks. Firstly, Cohen highlighted the modulations of ongoing theta (~6 Hz) power oscillations within the midfrontocentral (MFC) region. Secondly, Cohen pointed to increased theta synchronicity between the MFC region and the wider task-related regions (also see Cohen & Cavanagh, 2011; Nigbur, Ivanova, & Stürmer, 2011; Padrão, Rodriguez-Herreros, Pérez Zapata, & Rodriguez-Fornells, 2015; Pinner & Cavanagh, 2017; Zavala et al., 2016). Furthermore, functional magnetic resonance imaging (fMRI) research has demonstrated that the conflict stages of the Flanker, Stroop and Simon tasks all activate the same areas of anterior cingulate cortex (ACC) (for reviews, see Botvinick et al., 2001; Botvinick, Cohen, & Carter, 2004; Ridderinkhof, Ullsperger, Crone, & Nieuwenhuis, 2004). Therefore, evidence from response conflict tasks suggests that IPC is linked to an MFC-centred network of theta EEG activity and to the ACC.

Conversely, another form of conflict, goal conflict, is theorised to be distinct from IPC (Gray & McNaughton, 2000). Within goal conflict, a goal constitutes a situation–motivation compound (McNaughton et al., 2016). For example, a hungry (motivation) mouse in a maze that contains food (situation) may view approaching the food as a goal. Goal conflict can then occur between the pairs of goals that hold a similar level of appeal but are incompatible (Gray & McNaughton, 2000). For instance, if the food in the maze was accompanied by the scent of a predator (situation), then a wary mouse (motivation) may be faced with the conflicting goal of avoiding the predator. As such, the circumstances and experimental tasks that generate goal conflict are distinct from those that generate IPC.

The neurology of goal conflict is also thought to differ from that of IPC (McNaughton et al., 2016). Specifically, researchers have theorised that goal conflict is processed by a network of neural structures, known as the Behavioural Inhibition System (BIS) (Gray, 1982; Gray & McNaughton, 2000; McNaughton & Corr, 2004; McNaughton et al., 2016). The core of BIS is the septohippocampal system which is driven by the supramammillary nucleus (Gray & McNaughton, 2000; McNaughton et al., 2016). Much like IPC, theta EEG is thought to be an important part of goal conflict, as theta synchronises the structures of the BIS (Gray & McNaughton, 2000). However, unlike IPC, the regions and synchronicity networks of EEG that are involved in goal conflict are still largely uncertain. Therefore, the neurology of goal conflict appears to be largely distinct from that of IPC but is also, in terms of EEG, less well understood.

To our knowledge, only two laboratories have investigated EEG effects during goal conflict. The first conducted a series of studies using a go/no-go task that was modified to produce goal conflict (Moore, Gale, Morris, & Forrester, 2006, 2008; Moore, Mills, Marshman, & Corr, 2012). Specifically, Moore et al.’s (2006, 2008, 2012) tasks created goal conflict by altering the order or properties of instructional stimuli during the *anticipatory* phase of the task. In high goal-conflict trials, participants are led to *anticipate* a “go” instruction but, instead, receive a “no-go” instruction, creating goal conflict. During the high goal-conflict trials, theta (4–8 Hz) power (Moore et al., 2006) and coherence (Moore et al., 2006, 2012) increased across the scalp, relative to the low goal-conflict trials. Furthermore, in a follow-up study, the researchers confirmed that the goal-conflict EEG effects did not extend into the alpha range (8–12 Hz) (Moore et al., 2008). Therefore, Moore et al.’s (2006, 2008, 2012) findings suggest that goal conflict may have wider spatial characteristics than IPC but that it still involves the same EEG waveband of theta (4–6 Hz).

However, it is worth noting that Moore et al. (2006, 2008, 2012) did not include procedures to limit the effects of volume conductance. Volume conductance occurs as electrical signals, originating from the brain, pass through the skull to be later recorded via EEG at the scalp (Nunez & Srinivasan, 2006). In some cases, the activity is not passed directly up through the skull but, rather, is passed up and out, to spread to multiple regions (Nunez & Srinivasan, 2006). Activity that is volume conducted in this manner can be misinterpreted as increased EEG synchronicity between regions, potentially causing a considerable amount of distortion within coherence analyses (Khadem & Hossein-Zadeh, 2014). Therefore, some caution must be taken when interpreting the spatial characteristics of goal-conflict EEG from Moore et al.’s findings (2006, 2008, 2012).

The second laboratory to investigate goal conflict did so using a calibrated stop-signal task (SST) (McNaughton, Swart, Neo, Bates, & Glue, 2013; Neo, Thurlow, & McNaughton, 2011; Shadli, Glue, McIntosh, & McNaughton, 2015; Shadli, Smith, Glue, & McNaughton, 2016). Much like Moore et al.’s (2012) go/no-go task, the calibrated SST manipulates the timing of instructional stimuli in a way that balances the participants’ expectations of receiving either a go or stop signal. This balance of expectation during the anticipatory stage of the task creates goal conflict (Neo et al., 2011). However, unlike Moore et al.’s (2012) task, the timing of the instructional stimuli in the SST is calibrated to account for the reaction times and accuracy rate of each participant. As such, the task should create a more consistent level of goal conflict across participants compared to Moore et al.’s (2012) goal-conflict task.

Previous applications of the calibrated SST have already successfully demonstrated, and replicated, the increases in right frontal (RF) theta that are (a) present during goal conflict (Neo et al. 2011; McNaughton et al., 2013) and (b) reduced by anxiolytic drugs (Shadli et al., 2015, 2016). Certain investigations of the task have also observed wider frontal increases in theta power during goal conflict (Neo et al., 2011; Shadli et al., 2016). Furthermore, on three occasions, the goal-conflict activity spreads into the conventional human alpha range (8–12 Hz) (McNaughton et al., 2013; Shadli et al., 2015, 2016). Therefore, the findings of the calibrated SST may suggest that, unlike IPC EEG, goal-conflict EEG can be understood through 4- to 12-Hz RF and, sometimes, wider frontal power. These findings would also run contrary to Moore et al.’s (2006, 2008, 2012) observations of scalp-wide theta-restricted (4–8 Hz) goal-conflict effects.

However, it is important to note that the EEG signals produced by the calibrated SST have only once been analysed in areas outside of the frontal regions and have not yet been analysed using either coherence or using frequency bands outside of the 4- to 12-Hz range (McNaughton et al., 2013; Shadli et al., 2015, 2016). Therefore, it appears that, of the two laboratories working on EEG goal conflict, Moore et al. (2006, 2008, 2012) have developed the most comprehensive EEG recording methodology for goal conflict, with their investigations of scalp-wide power and coherence across multiple wavebands, whereas, Neo et al. (2011), McNaughton, Swart, Neo, Bates, and Glue (2013) and Shadli et al. (2015, 2016) have developed the most effective goal-conflict task with their calibrated SST. As such, it may be of great utility to combine the approaches of the two laboratories and to investigate the calibrated SST using a full scalp-wide topography of electrodes, a measure of coherence and a wide range of frequency bands. Doing so would significantly advance the literature on the EEG properties of goal conflict and would allow for further comparisons with the EEG properties of IPC.

## 1.1 The present study

Subsequently, in the present study, we will combine Moore et al.’s (2012) comprehensive EEG approach with the calibrated SST. Furthermore, we will take measures to attenuate the influence of problematic volume conductance (see Section 2.3.2), which may have distorted the coherence findings of Moore et al.’s (2006, 2008, 2012) work. In terms of predictions, we expect to observe the following changes during the high, compared with the low, goal-conflict condition of the calibrated SST. Firstly, we expect to observe increased MFC theta power (following Cohen, 2014; Cavanagh & Frank, 2014). Secondly, we expect to observe increased lateral frontal power within the 4- to 12-Hz range (following McNaughton et al., 2013; Neo et al., 2011; Shadli et al., 2015, 2016). Thirdly, we expect to observe increased theta (4–8 Hz) power in regions other than the MFC and lateral frontal regions (following Moore et al., 2006). Finally, we expect to observe increased theta coherence in region pairs across the scalp (following Moore et al., 2006, 2012).

## 2 Methods

### 2.1 Participants

Thirty-six healthy, right-handed participants (nine males), aged 18 to 42 years (*M* = 26.32; SD = 9.31) were recruited. Data from two female participants were excluded due to high levels of artefacts (for criteria, see Section 2.3.1). Ethical approval for the study was obtained from the University of Portsmouth Science Faculty Ethics Committee.

### 2.2 Procedure

Each participant completed four paper-based psychometric tests (for details, see Section 2.5) before being prepared for the EEG recording (see Section 2.3.1). Following preparation for EEG, the participant was seated in front of a laptop to complete an SST (Shadli et al., 2015). During the task, the participant monitored the screen for the appearance of a fixation circle. After 500 ms, an arrow, pointing left or right, appeared in the circle. The participant responded with a left or right mouse click using their right index finger as quickly as possible. These trials featured in the practice block, at the start of the task, and in the main part of the task, which followed. We refer to the 30 trials that appeared in the practice block as practice response trials and to the 288 trials that appeared in the main body of the task simply as go trials. Data from the 30 practice response trials were included in neither the EEG nor behavioural analyses and were only used for task timing calibration purposes (see below). In contrast, the go trials were included in EEG and behavioural analyses as well as in task timing calibration. After each response, feedback was administered in the form of an emoji.

**Figure 1.**
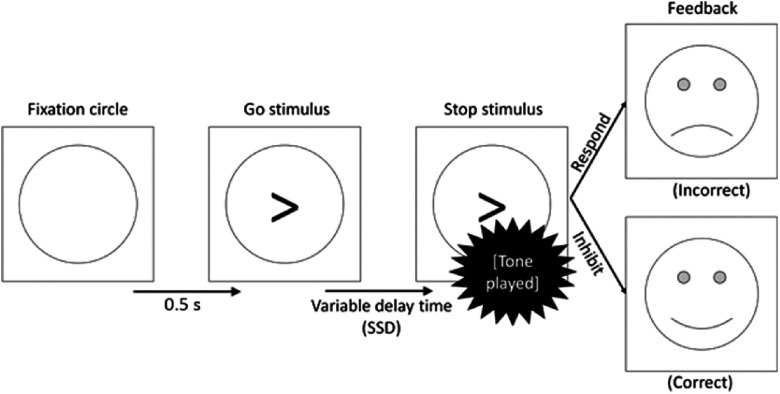
Flow diagram of a stop trial proceeding from left to right.

In addition to go trials, stop trials appeared 25% of the time (comprising 96 individual trials in total). No stop trials were included in the practice block. In these trials, the arrow was followed by an auditory tone of 1000 Hz for 0.5 s. This tone indicated that response should now be withheld (see Figure 1). The delay between the arrow and the tone, known as the *stop-signal delay* (SSD), varied in two respects. Firstly, there were three types of trial (short SSD, intermediate SSD and long SSD). This was thought to manipulate the level of goal conflict between the go and stop tendencies. Secondly, the exact delay in each of these conditions varied from participant to participant, depending on their reaction time and accuracy rate. It is in this sense that the task is calibrated to each participant to ensure that goal conflict occurs consistently across participants.

In short SSD trials, the tone followed the arrow almost immediately (delay = [mean reaction time to the previous 16 go trials] × 0.2). This provided the participant with plenty of time to process the stop signal, thus presenting a clear instruction to inhibit and causing low levels of goal conflict. Conversely, in the long SSD trials, the tone followed the arrow after a considerable delay (delay = [mean reaction time to the previous 16 go trials] × 0.8). This gave the participant very little time to process the stop signal and provided them with a clear (but misleading) signal to respond. Therefore, the long SSD trials should also create a low level of goal conflict.

Finally, the intermediate SSD trials used a staircase model for the delay. The staircase began at 45% of the participant’s mean reaction time in the 30 practice response trials and increased/decreased by 30 ms for every successful/unsuccessful inhibition, respectively. Therefore, the delay in this stop condition continually adjusted itself to produce a consistent level of goal conflict across participants and trials (but see the discussion of goal-conflict EEG effects differing across task blocks in Shadli et al., 2015). In behavioural terms, previous work on this task has suggested that participants should successfully inhibit their response in around 80% of the short SSD and around 20% of the long SSD trials (McNaughton et al., 2013; Neo et al., 2011; Shadli et al., 2015). However, accuracy rates for inhibiting should be nearer to 50% in the intermediate SSD trials, generating a higher degree of goal conflict between the, now equally weighted, goals. Overall, the task contained 384 trials (go: 288, stop short SSD: 32, stop intermediate SSD: 32, stop long SSD: 32).

### 2.3 Physiological measures

#### 2.3.1 EEG recording

**Figure 2.**
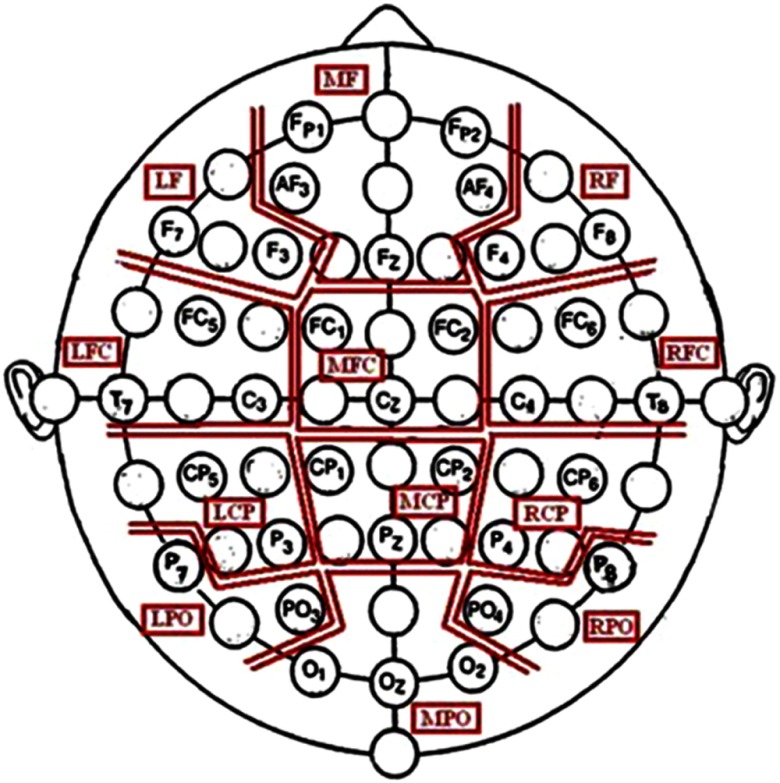
Outline of the ROIs that were based on Bosch, Mecklinger, and Friederici (2001). LCP, left centroparietal; LFC, left frontocentral; LPO, left parietooccipital; MCP, mid centroparietal; RCP, right centroparietal; RPO, right parietooccipital (figure taken, with permission, from Moore et al., 2012).

Continuous EEG was recorded from 32 scalp electrodes arranged in the 10–20 system using a Brain Vision QuickAmp (version 1.03.0004; Brain Products, Gilching, Germany) sampling at 2000 Hz. Electrodes were combined into 12 regions of interest (ROIs) (see Figure 2). Impedances were below 10 kΩ with anterior frontal z used as the subject ground. Horizontal and vertical electrooculogram activity was recorded using independent electrodes placed at the outer canthi of the eyes as well as above and below the right eye. The filters used consisted of a 0.531-Hz high-pass, a 70-Hz low-pass and a 50-Hz notch.

Data were analysed offline using Brain Analyzer (version 2.21; Brain Products, Gilching, Germany). Firstly, data were re-referenced to a common average reference. Next, eye movement artefacts were corrected using a BrainVision independent components analysis-based ocular artefact removal tool trained on data from the horizontal electrooculogram and vertical electrooculogram channels (Jung et al., 2000). Any epochs containing amplitudes outside of the range of – 75 to + 75 μV following ocular correction and epoching were marked for rejection. If more than 15% of a participant’s epochs were marked for rejection, then the participant’s data were not included in the EEG analyses.

#### 2.3.2 Controlling for volume conduction

The BrainVision current source density (CSD) transform was applied to the raw EEG data prior to epoching (see Section 2.4.1 for details of the epochs) using an “m” value (spine stiffness) of 4 and a lambda variable of 1e^−5^ (in line with Kayser & Tenke, 2015). This particular variation uses the formulas of Perrin, Pernier, Bertrand, and Echallier (1989) to produce surface Laplacian estimates^1^. Functionally, the CSD acts as a spatially driven band-pass filter. Specifically, the CSD takes into account the estimated physical distance between each electrode (based on the fractional distances of the 10–20 system) and the phase lag between the signals recorded at each electrode. By doing so, the CSD can: (a) for each electrode, produce a value to quantify the signal activity at the electrode, known as a surface Laplacian estimate and (b) identify signals that have spread from a single source to multiple electrodes due to volume conductance (i.e., signals that appear at multiple bordering electrodes with no perceivable phase lag). The CSD then attenuates any activity that appears to have resulted from this form of volume conductance (Kayser & Tenke, 2015). Controlling for volume conduction in this manner is particularly appropriate for analyses that examine the similarity of activity across multiple areas, such as coherence analysis (Khadem & Hossein-Zadeh, 2014). Furthermore, this version of the CSD transform has been demonstrated to work well in low electrode-density recordings, such as ours (Kayser & Tenke, 2006).

The surface Laplacian estimates produced by the CSD transform are effectively reference-free (Kayser & Tenke, 2015). More specifically, the reference that is applied to the EEG data, prior to CSD transform, does not affect the surface Laplacian estimates produced by the CSD^2^ (see Kayser & Tenke, 2015). Therefore, the CSD transform mitigates many of the biases that are inevitably introduced when using a referencing technique (see Kayser & Tenke, 2010). Finally, conducting a CSD transform changes the unit of measurement from µV to µV/m^2^ (see Tenke et al., 2017). Raw EEG data that have been cleaned and CSD transformed will be referred to as CSD EEG for the remainder of the methods section.

### 2.4 EEG data reduction

#### 2.4.1 Defining the high and low goal-conflict conditions

First, CSD EEG data relating to the three types of stop trial (i.e., short SSD, intermediate SSD and long SSD) were segmented to form 500-ms epochs beginning at the inhibitory tone played during the stop trials. Then, for each stop trial, the preceding go trial was identified and the corresponding epoch (i.e., with matching temporal characteristics) was extracted. This enabled identification of the epochs contributing to the go short, go intermediate and go long trials. Overall, this approach yielded 32 epochs for each type of stop trial (i.e., short SSD, intermediate SSD and long SSD) and, similarly, 32 epochs for each category of the corresponding go trials (i.e., go short, go intermediate and go long).

A fast Fourier transform was then applied to all 192 of the individual 500-ms epochs to derive both CSD power^3^ and coherence spectra associated with the full topography of electrodes (see Section 2.4.2 for details of CSD power and coherence spectra approach). Following this stage, the CSD power and coherence values of each of the go trials were subtracted from their corresponding stop trial yielding, per participant, a trial-by-trial change in CSD power and change in coherence spectra representation for the short, intermediate and long SSD trials. The subtraction step was included to isolate goal-conflict activity relating to the appearance of the stop signal (after Neo et al., 2011; Shadli et al., 2015, 2016)^4^.

Lastly, the CSD power and coherence spectra representations, following subtraction for the short, intermediate and long SSD trials, were averaged (respectively) across trials to form, for each participant, a single CSD power and coherence spectra representation of their response to each of the SSD types. The resultant data associated with intermediate SSD trials were then defined as the high goal-conflict condition, while the activity from the short and long SSDs were averaged to form the low goal-conflict condition^5^.

#### 2.4.2 Extraction of wavebands, CSD power/coherence spectra and ROIs

Trial-by-trial CSD power and coherence spectral data were extracted within the delta (1–4 Hz), low theta (4–6 Hz), high theta (6–8 Hz), low alpha (8–10 Hz), high alpha (10–12 Hz), low beta (12–20 Hz), high beta (20–30 Hz) and gamma (30–50 Hz) wavebands. Initially, to form the CSD power spectra, the CSD EEG data were transformed into complex CSD power (µV/m^2^)^2^ including a periodic Hamming window with a 20% taper. This produced a spectral decomposition with a frequency resolution of 2 Hz. The Hamming window was used, instead of a square window, to more effectively reduce the influence of signal distortion at the ends of each epoch and to emulate a more periodic signal. Then, to form the coherence spectra, smoothed waveband-specific cross CSD power spectra from pairs of electrodes (C_XY_) were derived using SSD trial type (i.e., short, intermediate and long SSDs as well as short, intermediate and long go, respectively) specific 500-ms epochs of artefact-free CSD EEG. These data were then entered into the following equation: *K*_*XY*_ = |*C*_*XY*_|2/(*C*_*XX*_ *C*_*YY*_), where *K*_*XY*_ is the resultant coherence value between the electrodes X and Y. This has the effect of normalising by the averaged powers of the signals at the compared electrodes (*C*_*XX*_ *C*_*YY*_) (for details, see Moore et al., 2012). The output value of such a coherence computation typically sits between 0 and 1 and represents the level of phase synchronicity between the involved electrodes. However, in the present study, we subtracted go activity from stop activity after the spectral transformations (see Section 2.4.1). Therefore, in this sense, we obtained a measure of change in coherence. As such, it is possible to obtain negative values in this instance, representing a reduction in coherence in the go trials relative to the stop trials.

CSD power and coherence spectral activity was then organised according to 12 ROIs (see Figure 2, Section 2.3.1). For the CSD power data, spectral activity was averaged among grouped electrodes for each ROI (see Andersen, Moore, Venables, & Corr, 2009; Moore et al., 2012). However, for the coherence data, activity in each of the 12 ROIs was considered in relation to each of the other 11 regions (which produced 66 ROI pairs). Specifically, the coherence level for each ROI pair was calculated by averaging the coherence levels of all possible inter-region coherence permutations (*see* Moore et al., 2012).

### 2.5 Behavioural and psychometric measures

#### 2.5.1 Behavioural measures

We recorded five behavioural measures in the present study. Firstly, we recorded the reaction time for the go trials. Secondly, we recorded the accuracy rates for the go trials. Finally, we recorded the accuracy rates for the short SSD, intermediate SSD and long SSD stop trials^6^. The go trial reaction time was defined as the median duration from the presentation of the arrow stimulus to the participant responding. The go trial accuracy rate was measured as the percentage of go trials in which the participant successfully executed a response. Stop trial accuracy rates were measured as the percentage of stop trials, in each SSD, respectively, in which the participant successfully inhibited a response. Additionally, the percentage of motor responses that were executed in trials associated with each of the SSD trial types was recorded.

#### 2.5.2 The Revised Reinforcement Sensitivity Questionnaire (Reuter, Cooper, Smillie, Markett, & Montag, 2015)

The Revised Reinforcement Sensitivity Questionnaire (r-RSTQ) is a self-report measure of reinforcement sensitivity theory (RST) that adheres to the revisions to the theory that fully differentiated the BIS from the other systems in RST (Gray & McNaughton, 2000). It features 31 items that are measured along a four-point Likert scale from “Strongly disagree” to “Strongly agree”. Of the 31 items, 11 contribute to the measure of Revised BIS (rBIS), 12 to the Fight Flight Freeze System and 8 to the Behavioural Activation System (for an overview of the systems, see Corr, 2008). The following is an example of an rBIS question: “If I have the choice between two appealing options, I have difficulty deciding on one”. For the purposes of this study, only results relating to the rBIS factor were entered for analysis.

#### 2.5.3 The State-Trait Anxiety Inventory (Spielberger, 1983)

Form “Y” from the State-Trait Anxiety Inventory (STAI) was used for this study. Form Y features 40 questions, half of which measure state anxiety and half of which measure trait anxiety. As these are conventionally presented to the participant together, data were recorded from both state and trait items; however, only data from the trait items were entered for analyses. The scale features a four-point Likert scale ranging from “Almost never” to “Almost always”. The STAI is mainly concerned with anxiety but also includes items measuring depression. Here, the Bieling, Antony, and Swinson (1998) STAI anxiety subscale was used. This updated subscale included items, from the STAI, that are related to anxiety and worry and excluded items that are related to sadness and self-depreciation.

#### 2.5.4 The Eysenck Personality Questionnaire Revised (Eysenck, Eysenck, & Barrett, 1985)

The Eysenck Personality Questionnaire Revised (EPQ-r) is a measure of biologically derived dimensions of personality. The dimensions of the scale heavily influenced the factor structure of RST (Gray, 1982). This version includes the dimensions of introversion–extraversion, neuroticism and psychoticism and features 100 yes/no items. For the purposes of this study, only results from the neuroticism factor were entered into the analysis.

#### 2.5.5 The Carver and White BIS/BAS scales (BIS/BAS scales) (Carver & White, 1994)

The BIS/behavioural activation system (BAS) scales are ubiquitous measures of RST. The scales feature 24 questions which are answered from 1 (“very true for me”) to 4 (“very untrue for me”). Of the 24, 4 relate to BAS Drive, 4 to BAS Fun-Seeking, 5 to BAS Reward Responsiveness, 7 to the BIS and 4 to filler material. As the measure was designed prior to the revised RST (Gray & McNaughton, 2000), it is necessary to separate items in the BIS scale pertaining to anxiety (BIS-anxiety) and to fear (BIS-fear). This was accomplished using the Heym, Ferguson, and Lawrence (2008) BIS-anxiety subscale. The following is an example of a trait BIS-anxiety question from the BIS-anxiety subscale: “I feel worried when I think I have done poorly at something important”. Only BIS-anxiety scores for each participant were entered for analysis.

### 2.6 Statistical analysis

#### 2.6.1 Analyses of variance of MFC and lateral frontal theta CSD power effects

Ten pairwise repeated measures analyses of variance (ANOVAs) were used to examine whether CSD power significantly increased in the MFC, RF and left frontal (LF) regions during the high, compared with the low, goal-conflict condition. Each ANOVA contained one factor: Condition (two levels: high conflict and low conflict). Separate ANOVAs were conducted for low theta (4–6 Hz) and high theta (6–8 Hz), low alpha (8–10 Hz) and high alpha (10–12 Hz) wavebands. The MFC region was only investigated in the low and high theta wavebands, as there is no precedent on which to justify a priori alpha examinations of this region. Conversely, McNaughton et al. (2013) and Shadli et al. (2015, 2016) have identified lateral frontal goal-conflict effects across the 4- to 12-Hz range, thus justifying theta and alpha a priori examinations of the LF and RF regions. Benjamini–Hochberg corrections for multiple comparisons were applied based on the 10 pairwise ANOVAs conducted (Benjamini & Hochberg, 1995; Blume et al., 2015; McDonald, 2008).

#### 2.6.2 Hierarchical ANOVAs of wider CSD power and coherence effects

Hierarchical, or nested, repeated measures ANOVA models were employed to test for differences in spectral EEG between the high goal-conflict and low goal-conflict conditions (following Bosch, Mecklinger, & Friederici, 2001; Hanslmayr et al., 2008; Moore et al., 2012). In doing so, data were initially entered into superordinate ANOVAs. Because we predicted that activity would occur within a particular waveband, namely theta, a separate superordinate ANOVA was conducted for each of the eight wavebands. Additionally, CSD power and coherence data were analysed in separate superordinate ANOVAs, making a total of 16 superordinate ANOVAs. Each superordinate ANOVA included the following factors: Condition (2 levels: low goal conflict and high goal conflict) and ROI (12 levels for CSD power ANOVAs and 66 levels for coherence ANOVAs) (see Section 2.3.1). Any interaction effects identified by these ANOVAs were followed up with subordinate ANOVAs to further search for significant results. Following Luck and Gaspelin (2017), we only report and follow up on the factors which are most pertinent to our research hypotheses. As such, a significant interaction or significant main effect involving the condition factor was required in the superordinate stage.

For the CSD power data, a natural log transformation was applied to normalise the distribution of the data prior to entry into a hierarchical ANOVA. The normalised CSD power data were then entered into a repeated measures superordinate ANOVA for each waveband. For the coherence data, a Fisher-*Z* transformation was applied (following Sarnthein, Petsche, Rappelsberger, Shaw, & von Stein, 1998; also see Halliday & Rosenberg, 2000) to normalise the data distribution. The Fisher-Z transform was applied to each coherence value individually using the following formula: [*z* = (ln (1 + *r*) – ln (1 − *r*))/2], where ln is the natural log and *r* is the coherence value. The normalised coherence data were then entered into a repeated measures superordinate ANOVA for each waveband^7^.

Benjamini–Hochberg corrections for multiple comparisons (Benjamini & Hochberg, 1995; Blume et al., 2015; McDonald, 2008) were applied at each stage of the hierarchical ANOVAs based on the total number of subordinate ANOVAs conducted at that stage. To deal with violations of sphericity, tests with more than two degrees of freedom were corrected using the Greenhouse Geisser (Abdi, 2010) epsilon value (reported as EPS) to adjust the degrees of freedom and resulting *p*-values. Uncorrected degrees of freedom, corrected *p*-values and the Greenhouse Geisser EPS values are reported for all such tests. Finally, partial-eta-squared (*ηp* ^2^) was reported as a measure of effect size that represents the proportion of the variance accounted for by the variable in question.

#### 2.6.3 Regression analyses of spectral EEG and psychometric data

For any ROIs and ROI pairs that showed differences in theta between the goal-conflict conditions during the ANOVAs, we examined whether high goal-conflict spectral EEG from those same ROIs and ROI pairs could be used to predict psychometrically measured traits using forced entry regression analyses. However, one of the assumptions of regression analyses is that the predictor variables are orthogonal, i.e., they are independent and do not share variance (Salkind, 2010), which is often not the case with EEG data (van Den Broek, Reinders, Donderwinkel, & Peters, 1998). Therefore, prior to being entered into the regression analyses, the data from the theta ROIs and ROI pairs (specifically, those which differed between the conflict conditions) were first entered into spatial principal component analyses (PCAs) to be grouped into orthogonal spatial components. The spatial components were each represented by a single, per-participant, value. The value was formed by combining spectral EEG activity from ROIs and ROI pairs contributing to the spatial component (see the following paragraph for details). These spatial components were then used as predictor variables in the regression analyses.

Specifically, high conflict theta data from each of the ROIs or ROI pairs which differed between the conflict conditions were entered into a spatial PCA. Principle components and variable loadings were calculated using the MATLAB “pca” function. The PCA computed a co-variance matrix and was therefore equivalent to singular value decomposition (Ferree, Brier, Hart, & Kraut, 2009). Singular value decomposition is particularly appropriate for high-dimensionality data sets, such as our EEG data set (Dien, Beal, & Berg, 2005; Duffy & Als, 2012; Kayser & Tenke, 2003). The number of spatial components was selected with parallel analysis, which reduces the risk of over-selecting components (Franklin, Gibson, Robertson, Pohlmann, & Fralish, 1995). For each spatial component, any variables (ROIs or ROI pairs) loading with a coefficient value of .3 or above (following Costello & Osborne, 2005), after varimax rotation (Abdi, 2010), were retained for the spatial component. To form a single value (component score) for each spatial component (for each participant), the centred spectral EEG data from its retained variables (ROIs or ROI pairs) were linearly combined with each variable weighted by its loading coefficient following rotation (following Brier et al., 2010; Duffy & Als, 2012).

The component scores of the spatial components were then entered together as predictor variables in forced entry regression analyses with psychometric scores used as independent variables. Specifically, regression analyses were conducted to predict each of the four psychometric variables (see Section 2.5). Therefore, four regressions were computed in total. As they were forced entry regression analyses, only one model, containing the component scores of all of the retained spatial components, was used in each regression analysis. The results were then corrected for multiple comparisons, based on the four analyses conducted, using Benjamini–Hochberg corrections (Benjamini & Hochberg, 1995; McDonald, 2008).

## 3 Results

### 3.1 Behavioural data

For the go trials, the median recorded reaction time was (*Mdn*: 441 ms; SE: 8 ms). The accuracy rate for go trials was 99%, demonstrating that participants adequately engaged with the task. There was an accuracy rate of 57% (SE = 1.36) in the intermediate SSD trials, which indicates that the overall motivation of participants, in these trials, was weighted slightly more towards inhibiting than responding. However, it was considerably more matched than both the short SSD (*M* = 93%; SE = 1.19) and long SSD (*M* = 19%; SE = 1.82) trial types, indicating a higher level of goal conflict during the intermediate SSD trials. In terms of overt motor response, these data indicate that, in the high conflict condition, a motor response was executed in 43% (calculated as 100% minus the 57% inhibition-accuracy rate) of the trials comprising the high goal-conflict condition. Conversely, in the low goal-conflict condition, the overall motor response rate was 44%. Specifically, the low goal-conflict condition overt motor response rate was calculated as: ((100% – 93% short SSD inhibition-accuracy rate) + (100% – 19% long SSD inhibition-accuracy rate))/ 2.

### 3.2 CSD power effects during goal conflict

#### 3.2.1 MFC theta (4–6 Hz) CSD power increased significantly during goal conflict

The pairwise ANOVA for MFC low theta CSD power revealed a significant increase in the high (*M* = 1.76, SE = 0.32), compared with the low (*M* = 0.76, SE = 0.21), goal-conflict condition, *F*(1,33) = 6.698, *p* < .05, *ηp* ^2^ = 0.169. Conversely, in the ANOVA for MFC high theta CSD power, there was no statistically detectable effect of goal conflict, *F*(1,33) = 3.447, *p* = .072, ns.

#### 3.2.2 No wider changes in CSD power were observed

The low theta, high theta, low alpha and high alpha ANOVAs for the LF and RF regions did not identify any statistically detectable effects of condition. We have included the ANOVA statistics for each comparison in Table 1, given that we included these comparisons in our hypotheses. Finally, none of the eight CSD power superordinate ANOVAs, which examined changes in CSD power across the scalp for each of the eight wavebands, identified any statistically detectable effects of condition nor of condition × ROI. Overall, therefore, the only significant change in CSD power observed during goal conflict was an increase in MFC theta CSD power.

**Table 1.** ANOVA statistics for the pairwise analyses of the lateral frontal regions between the high and low goal-conflict conditions within the theta and alpha wavebands

Region	Waveband	Df	df error	*F*	*p*	*ηp* ^2^
RF	Low theta	1	33	0.190	.666	0.006
	High theta	1	33	0.226	.142	0.007
	Low alpha	1	33	0.001	.972	<.001
	High alpha	1	33	0.534	.470	0.016
LF	Low theta	1	33	1.963	.170	0.056
	High theta	1	33	0.617	.438	0.019
	Low alpha	1	33	1.261	.269	0.037
	High alpha	1	33	0.798	.378	0.024

### 3.3 Coherence effects during goal conflict

#### 3.3.1 Increased high theta (6–8 Hz) coherence during conflict

**Figure 3.**
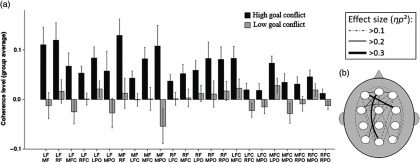
(a) Changes in high theta (6–8 Hz) coherence levels in the high and low goal-conflict conditions. Of particular note is that every change in theta occurs as an increase in theta during the high, compared with the low, goal-conflict condition. (b) Effect sizes of the increases in high theta coherence during the high, compared with low, goal-conflict condition (*N* = 34). In general, theta coherence can be observed across most of the scalp. In particular, the strongest effects occur between the MF–MPO and LF–RFC region pairs. LFC, left frontocentral; LPO, left parietooccipital; RPO, right parietooccipital

The superordinate ANOVA for high theta revealed a significant interaction effect of condition × ROI (*F*(65,2145) = 3.10, *p* < .01, EPS = 0.093). This justified the use of a subordinate ANOVA for each ROI pair. The subordinate ANOVAs revealed increases in coherence during the high goal-conflict, relative to the low goal-conflict, condition among 23 pairs of regions (see Table 2 for details). These data have also been depicted in Figure 3a, showing the extent to which high theta coherence increases during high goal-conflict, relative to low goal-conflict, trials. Additionally, the strength of the effects between ROIs for high theta is shown in Figure 3b (topographical map). Of particular note, the largest effect sizes are found between the LF-right frontocentral (RFC) ROI pairs and between the mid frontal–mid parietooccipital (MF–MPO) ROIs. The superordinate ANOVAs for the low theta, low alpha, high alpha, low beta, high beta and gamma wavebands did not identify any statistically detectable effects of goal conflict. Subsequently, no follow-up analyses were carried out on coherence data associated with those wavebands.

**Table 2.** ANOVA and descriptive statistics for the coherence pairs showing significant increases in the high theta band during the high, compared with the low, goal-conflict condition

Coherence pair	*F*(1,33)	*p*	*η*_*p*_^2^
LF and MF	8.38	<.01	.202
LF and RF	7.56	<.05	.186
LF and MFC	6.86	<.05	.172
LF and RFC	16.30	<.001	.332
LF and LPO	5.79	<.05	.149
LF and MPO	4.49	<.05	.120
MF and RF	9.50	<.005	.223
MF and LFC	8.23	<.01	.200
MF and MFC	8.01	<.01	.195
MF and MPO	19.51	<.001	.372
RF and LFC	4.76	<.05	.126
RF and MFC	4.15	<.05	.112
RF and LPO	4.39	<.05	.117
RF and MPO	4.76	<.05	.126
RF and RPO	5.14	<.05	.135
LFC and MFC	4.40	<.05	.118
LFC and RFC	8.28	<.01	.201
LFC and MPO	7.66	<.01	.188
MFC and LPO	8.28	<.01	.201
MFC and MPO	7.66	<.01	.188
MFC and RPO	4.72	<.05	.125
RFC and MPO	4.46	<.05	.119
RFC and RPO	4.93	<.05	.130

LPO, left parietooccipital; LFC, left frontocentral; RPO, right parietooccipital.

#### 3.3.2 Increased delta (1–4 Hz) coherence during conflict

The superordinate ANOVA for delta revealed a significant interaction of condition × ROI (*F*(65,2145) = 2.78, *p* < .001, EPS = 0.92). We subsequently conducted a subordinate ANOVA for each ROI pair. A single ROI pair, involving the LF and MF ROIs, demonstrated a significant increase in delta coherence during the high (*M* = 0.16, SE=0.04), compared with the low (*M* = 0.07, SE = 0.03), goal-conflict condition (*F*(1,33) = 13.64, *p* < .005, *ηp* ^2^ = 0.251).

### 3.3 High goal-conflict EEG activity predicts neuroticism in forced entry regression models

#### 3.3.1 Generation of orthogonal components via PCA prior to entry into regression analyses

**Figure 4.**
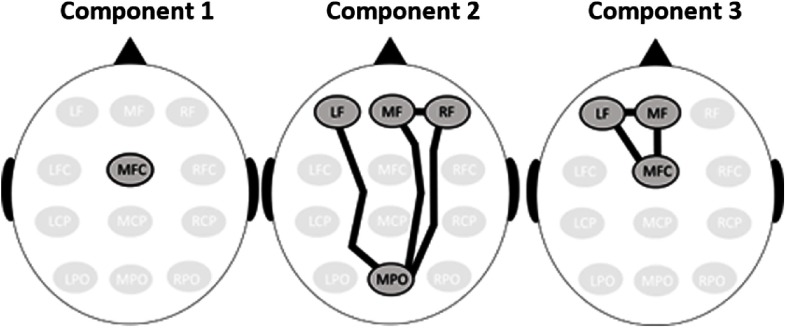
Spatial components produced by a PCA of high goal-conflict theta EEG. Only regions or region pairs which differed significantly between the high and low goal-conflict conditions in the ANOVAs were entered into the PCA.

High goal-conflict spectral EEG activity from the 23 theta ROI pairs (see Table 2) and the MFC region was entered into the spatial PCA (see Section 2.6.3). These region pairs and this region were included in the PCA because they differed significantly between the high and low goal-conflict conditions in the ANOVA analyses. Based on the result of the parallel analysis, we adopted a three-component solution that explained 96% of the total variance. The first identified spatial component involved the MF CSD power alone. The second component involved the LF–MPO, MF–RF, MF–MPO and the RF–MPO ROI pairs. The final component involved the LF–MF, LF–MFC and the MF–MFC ROI pairs (see Section 2.3.1 for abbreviation definitions). Topographic summaries of these components can be found in Figure 4.

#### 3.3.2 Orthogonal high goal-conflict components predict neuroticism in regression analyses

Component scores for the three orthogonal components were simultaneously entered as predictor variables into four forced entry regression analyses. In predicting trait neuroticism, the regression model was significant (*R*^2^ = .278, *F*(3,29) = 3.720, *p* < .05). However, while the overall model was significant, only component 3 significantly contributed to the model. Specifically, component 3 showed a significant negative association with neuroticism. The other two components (1 and 2) were non-significantly, but positively, associated with neuroticism (see Table 3). The regression models predicting trait STAI-anxiety (*R* ^2^ = .134, *F*(3,26) = 1.337, *p* = .284, ns), BIS-anxiety (*R* ^2^ = .135, *F*(3,31) = 0.574, *p* = .454, ns) and rBIS (*R* ^2^ = .009, *F*(3,31) = 0.291, *p* = .593, ns) were non-significant (for beta weights, zero-order and partial correlations, see Table 3).

**Table 3.** Coefficients and model values resulting from the neuroticism regression analysis.

	Neuroticism (EPQ-r)			rBIS (r-RSTQ)			Trait Anxiety (STAI)			BIS (BIS/BAS scales)		
	C 1	C 2	C 3	C 1	C 2	C 3	C 1	C 2	C 3	C 1	C 2	C 3
*ß*	0.125	3.38	−15.76	0.290	0.968	−0.697	0.223	2.669	−7.584	0.195	0.099	0.291
*t*	0.287	0.873	−3.107	0.545	0.218	−1.137	0.620	0.834	−1.874	0.729	0.044	0.094
*p*	.776	.39	<.005	.590	.829	.265	.541	.412	.070	.472	.965	.926
ZO	0.032	0.121	−0.367	0.097	−0.080	−0.216	0.106	−0.045	−0.316	0.135	0.026	0.028
Partial	0.057	0.376	−0.516	0.101	0.041	−0.207	0.123	0.165	−0.354	0.134	0.008	0.018

*ß*, beta coefficient; C, Component; *p*, *p* value given as the predictor’s significance within the model; partial, partial correlation value; *t*, *t* value used to calculate the predictor’s significance within the model; ZO, zero-order correlation value.

## 4 Discussion

In the present study, we combined Moore et al.’s (2012) comprehensive EEG recording methodology with Shadli et al.’s (2015) calibrated SST task to advance research into goal-conflict EEG. Specifically, we investigated scalp-wide CSD power and coherence during the calibrated SST within a wide range of wavebands. From our results, we can identify five main findings. Firstly, as predicted, MFC theta CSD power increased during the goal-conflict phase of the calibrated SST. Secondly, contrary to our hypotheses, EEG CSD power did not change outside of the MFC during goal conflict. Thirdly, as predicted, we observed scalp-wide increases in theta coherence during the goal-conflict phase of the calibrated SST. Fourthly, in addition to our predictions, we observed a single increase in frontal delta coherence. Finally, PCA components formed from high goal-conflict EEG data significantly predicted trait neuroticism. Taken together, our findings demonstrate that goal conflict involves a network of MFC theta (4–6 Hz) CSD power, scalp-wide theta (6–8 Hz) coherence and frontal delta (1–4 Hz) coherence. In the following sections, we will comment on each of these findings to provide comparisons with IPC EEG and with previous work on goal-conflict EEG.

### 4.1 Goal conflict was effectively manipulated by the task

The behavioural findings suggest that the calibrated SST effectively manipulated goal conflict. Specifically, the mean accuracy rate in the trials that contributed to the high goal-conflict condition neared 50%. This suggests that the levels of anticipation for receiving either a go or stop signal were near equally weighted in these trials. Subsequently, a high degree of goal conflict should have been created across the participants in the high goal-conflict condition. Conversely, in the trials contributing to the low goal-conflict condition, accuracy rates were much further from 50%. Therefore, as expected, the high goal-conflict condition does appear to have created a higher degree of goal conflict than the low goal-conflict condition. Our behavioural results are highly similar to those of previous studies using this task (McNaughton et al., 2013; Neo et al., 2011; Shadli et al., 2015).

#### 4.2.1 The only change in CSD power during goal conflict was an increase in MFC theta (4–6 Hz)

As predicted, MFC theta CSD power increased during the high, compared with the low, goal-conflict condition. This was the only significant change in CSD power during goal conflict. Therefore, our findings suggest that MFC theta is the primary CSD power correlate of goal conflict. This presents an interesting contradiction to previous investigations of goal-conflict EEG (McNaughton et al., 2013; Moore et al., 2006, 2012; Neo et al., 2011; Shadli et al., 2015, 2016) and a striking similarity to IPC EEG (Cavanagh & Frank, 2014; Cohen, 2014). In the following section, we shall discuss each of these comparisons in detail.

Using their modified go/no-go task, Moore et al. (2006) identified scalp-wide increases in theta (4–6 Hz) power during goal conflict. Therefore, our CSD power findings appear to contradict those of Moore et al. (2006). Specifically, we identified increased theta CSD power that, far from being scalp-wide, was limited to the MFC region. However, it is worth noting that a replication of Moore et al.’s (2006) task later identified no significant changes in power during the goal-conflict stage of the task (Moore et al., 2012). Therefore, changes in scalp-wide theta power during goal conflict have yet to be replicated. Furthermore, the present increase in MFC theta emerged from, what we propose is, a more effective goal-conflict task. Specifically, the task used in the present study included a calibration mechanism that advances Moore et al.’s (2006, 2012) go/no-go task by ensuring that the level of goal conflict created was consistent across participants. Therefore, the increase in MFC theta CSD power during goal conflict, observed here, may be a more accurate summary of region-specific goal-conflict EEG than Moore et al.’s (2006) scalp-wide increases in theta power.

Additionally, our observation of increased MFC theta CSD power during goal conflict is a unique finding among previously reported outcomes for the calibrated SST (i.e., across McNaughton et al., 2013; Neo et al., 2011; Shadli et al., 2015, 2016). Specifically, previous investigations of this task have identified increased lateral frontal theta (4–12 Hz) power during goal conflict (McNaughton et al., 2013; Neo et al., 2011; Shadli et al., 2015, 2016). Only one previous calibrated SST investigation has analysed goal-conflict activity within the MFC region (Neo et al., 2011). Therefore, our observation of increased MFC theta CSD power adds to previous research by identifying a new region that may be related to goal conflict. However, at the same time, we were not able to replicate the changes in lateral frontal theta power that has been replicated in multiple previous applications of the task (McNaughton et al., 2013; Neo et al., 2011; Shadli et al., 2015, 2016). Therefore, our findings run contrary to a great deal of previous work by suggesting that changes in localised EEG during goal conflict are restricted to the MFC.

Furthermore, our findings also disagree with previous examinations of the SST in terms of the wavebands involved. Specifically, our region-specific (i.e., non-connectivity) EEG findings were restricted to the 4- to 6-Hz waveband and not to the 4- to 12-Hz waveband range identified in the previous work (McNaughton et al., 2013; Shadli et al., 2015, 2016). McNaughton et al. (2013) explained that the wide waveband range, observed in their findings, may be due to the origins of the theta activity. Specifically, they suggested that their goal-conflict EEG activity may have spread into, what is conventionally considered to be, the human alpha range because it has become synchronised with hippocampal theta which, in rodents, occurs in the 4- to 12-Hz range (see Young & McNaughton, 2009). Therefore, McNaughton et al. (2013) seem to suggest that, when discussing goal conflict, “theta” (as in, hippocampal-locked theta) may need to be interpreted beyond its conventional human definition of 4 to 8 Hz. Conversely, in the present study, it appears that region-specific (i.e., non-connectivity) goal-conflict EEG is, in fact, limited to the conventional human theta range and not to the extended 4–12 Hz rodent theta range.

However, there is a crucial consideration to made here which may explain why our localised EEG findings appear to contradict previous research so consistently. Specifically, previous examinations of goal-conflict EEG have recorded measures of power (μV^2^) (McNaughton et al., 2013; Moore et al., 2006, 2012; Neo et al., 2011; Shadli et al., 2015, 2016). Conversely, in the present study, we recorded a measure of CSD power (μV/m^2^)^2^. The CSD transformation attenuates volume conductance, enabling the accurate identification of connectivity measures and making it of considerable benefit to the present study (Khadem & Hossein-Zadeh, 2014). However, as mentioned, all EEG is volume conducted to some extent (Nunez & Srinivasan, 2006). Therefore, the job of the CSD transform is to determine which forms of volume conductance are problematic, which would distort the connectivity analyses and which forms of volume conductance are acceptable (Kayser & Tenke, 2015). This then, of course, becomes a question of what is considered problematic or acceptable volume conductance. In trying to decide between the two, the CSD transform that we applied here has been shown to be effective at reducing connectivity–analysis–distorting activity across medium and long range pairs (Kayser & Tenke, 2015). However, in doing so, the CSD transform can also attenuate localised EEG activity that originates from deep cortical sources (Kayser & Tenke, 2015). Therefore, the CSD transform can produce more accurate connectivity results (i.e., effects between the pairs of regions) but may hide certain localised results (i.e., effects at individual regions). Subsequently, if the lateral frontal and wider theta power effects, observed in previous studies (McNaughton et al., 2013; Moore et al., 2006, 2012; Neo et al., 2011; Shadli et al., 2015, 2016), are derived, even in part, from deep cortical sources, then they may have been attenuated by the CSD transform. Therefore, lateral frontal, and even scalp-wide, goal-conflict effects may be visible in measures of power but not CSD power. Future research may wish to investigate this possibility further by computing both power and CSD power from a single calibrated SST data set.

Finally, it is worth mentioning that our localised EEG findings appear to overlap with those of IPC research. Specifically, both the goal-conflict-related CSD power effects of the present study and the IPC-related power effects of previous studies appear to be focused around the MFC region (see Cohen, 2014). Therefore, when only localised (non-connectivity) activity is considered, goal conflict and IPC appear to be highly similar in terms of EEG. This may suggest that there is also some similarity between the underlying source activity of goal conflict and IPC EEG. In particular, fMRI research has linked IPC conflict to the ACC (Botvinick et al., 2001; Botvinick et al., 2004; Ridderinkhof et al., 2004). The ACC is known to be a generator of midline theta (Asada, Fukuda, Tsunoda, Yamaguchi, & Tonoike, 1999; Ishii et al., 1999; Onton, Delorme, & Makeig, 2005). This may explain why IPC is so commonly linked to modulations of midline theta EEG. It may, therefore, also be possible that the ACC is generating the mid-central theta that we observed here during goal conflict. Furthermore, Young and McNaughton (2009) have noted that the ACC can display hippocampal-locked theta from its rostral–rostral and caudal subsections and hippocampal-independent theta from its wider subsections. This is important because the hippocampal system is the core of the BIS (Gray & McNaughton, 2000) but is thought to be largely unrelated to IPC. Therefore, the observation of increased MFC theta in both IPC (Cohen, 2014) and in goal conflict may suggest that the ACC is the source of both effects but that the IPC and goal-conflict effects are generated by separate subdivisions of the ACC. However, this is a highly tentative conclusion that requires a great deal of further research. For now, we have identified that increased MFC theta is common across both goal conflict and IPC.

#### 4.2.2 Theta coherence increases across the scalp

Coherence is a measure of the similarity or synchronicity of frequency-domain EEG between multiple regions of the scalp (Lachaux, Rodriguez, Martinerie, & Varela, 1999). Therefore, the pairs of regions that show increased levels of coherence during goal conflict may be tentatively speculated to be displaying functional connectivity and, subsequently, to be part of a goal-conflict network. In the present study, increases in theta coherence were identified across the scalp during the high goal-conflict condition of the calibrated SST. These effects were identified by a hierarchical ANOVA ending with alpha-corrected pairwise subordinate ANOVAs. As such, the coherence findings emerged despite their analyses being more conservative than those of the CSD power EEG. This suggests that coherence, rather than CSD power, may provide the most easily identifiable non-MFC effects for future studies of calibrated goal-conflict tasks. Our own coherence results exhibited four main characteristics. Firstly, changes in theta coherence occurred predominantly within the theta (6–8 Hz) waveband. Secondly, changes in theta coherence were universally the increases in coherence. Thirdly, the MF–MPO and LF–RFC region pairs displayed the strongest effects. Finally, the majority of the coherence increases could be considered to be small effects.

The observation of predominantly *theta* increases in coherence during goal conflict aligns with the predictions of BIS-driven activity. Specifically, the structures of the BIS are thought to be synchronised by hippocampal-locked theta activity (Gray & McNaughton, 2000). Additionally, researchers have also proposed that BIS-directed theta should be visible to EEG recordings during goal conflict (Moore et al., 2006). Therefore, the goal-conflict-related increases in theta coherence observed in the present study align with Gray and McNaughton’s (2000) and Moore et al.’s (2006) predictions. Specifically, we have identified a network of activity that may be driven by the BIS.

Within this network, the largest changes in theta involved the MF–MPO and LF–RFC region pairs. To make sense of the MF–MPO region pair, we will examine the possible role of the MF and MPO separately. Firstly, the involvement of the MF region suggests that the activity may be part of the conflict resolution and inhibitory functions of the BIS. Specifically, magnetoencephalogram research has linked medial prefrontal theta to interference resolution and inhibition during episodic memory retrieval (Ferreira, Marful, Staudigl, Bajo, & Hanslmayr, 2014). Specifically, during memory recall, the hippocampus (acting as the core of the BIS) is thought to reduce the effects of interference by eliminating competing memories to enable the individual to recall only the correct memory (Gray & McNaughton, 2000). Furthermore, rodent research has identified increased theta synchrony between the medial prefrontal cortex and the hippocampus during memory retrieval (O'Neill, Gordon, & Sigurdsson, 2013) and during the encoding of behaviourally relevant events (Jarovi, Volle, Yu, Guan, & Takehara-Nishiuchi, 2018). Therefore, it is possible that the MF theta, observed here as part of an MF–MPO region pair during goal conflict, is related to the BIS process of resolving conflicts between competing, behaviourally relevant, memories during recall. The MPO part of the MF–MPO region pair may relate to sensory processing. Specifically, hippocampal–frontal–occipital networks have been previously implicated in sensory processing (Sehatpour et al., 2008). Therefore, the MF part of the region pair may signal the involvement of conflict resolution between memories, and the MPO part of the region pair may specify that the memories in question relate to sensory information. Unfortunately, it is less clear as to why LF–RFC coherence increased during goal conflict with such a large effect size.

In addition to the large effects, a multitude of moderate and small effects were also observed. Specifically, of the 23 increases in theta coherence during goal conflict, 6 presented as moderately sized effects and 15 presented as small-sized effects. Brunner, Billinger, Seeber, Mullen, and Makeig (2016) demonstrated that low levels of residual volume conductance can remain between pairs of neighbouring regions following the application of a CSD. Therefore, interpreting the small changes in coherence during conflict within each ROI pair individually may not be appropriate. Rather, it is important to notice the trend provided by these changes in coherence, specifically, that they are all increases in theta coherence and that they occur across the scalp. As such, our results support those of previous studies that identified scalp-wide increases in theta coherence during goal conflict (Moore et al., 2006, 2012).

In comparison to coherence changes during IPC, coherence changes during goal conflict appear to operate over a much wider set of region pairs. Specifically, IPC has been linked to a network of increased theta centred around the MFC. For example, in their reviews, both Cavanagh and Frank (2014) and Cohen (2014) highlighted the finding of IPC-related increases in theta between the MFC and (a) the parietal regions, (b) the lateral frontal regions and (c) the occipital regions. Conversely, in the present study, we identified goal-conflict-related increases in theta coherence across the scalp with no obvious centre point of the coherence network. Therefore, coherence metrics can present a clear distinction between the neural processing of IPC and goal conflict.

Interestingly, the centroparietal regions were the only regions that were not involved in coherence changes during goal conflict. Previous IPC tasks have identified links between the MFC and parietal regions on multiple occasions (Nigbur, Cohen, Ridderinkhof, & Sturmer, 2012; van de Vijver, Ridderinkhof, & Cohen, 2011). Therefore, the involvement or non-involvement of parietal coherence may be another crucial difference between goal conflict and IPC. Alternatively, this discrepancy may result from the way that the SST controls for motor activity, which is a function of the parietal cortices (Cavanagh & Frank, 2014), compared to other conflict tasks. For example, Nigbur et al. (2012) observed increased coherence between the MFC and left-parietal cortex during right-handed response inhibitions in an IPC task. Additionally, when the same conflict task required a left-handed response inhibition, increased coherence was observed between the MFC and the right-parietal cortex. Therefore, MFC-parietal coherence appears to be directly related to changes in motor response processing. However, in the present study, the motor response rates were nearly identical in the low and high conflict conditions (45% and 43%, respectively). Furthermore, participants responded with their right hand in all trials. Subsequently, there were no differences in response rates or response laterality between our conditions. This may explain why, contrary to previous studies of conflict tasks, our results did not involve the parietal cortex. Either way, our results suggest that there are dramatic differences in the coherence networks involved in IPC and goal conflict.

#### 4.2.3 Frontal delta coherence and conflict

A single increase in LF–MF delta during goal conflict resulted from a superordinate ANOVA followed by subordinate pairwise ANOVAs. Given the close proximity of the two regions, the increase may have been influenced by volume conduction (Rutkove, 2007). However, if this was the case then we might also expect to see a large change in delta CSD power in these regions, indicating a strong source that spreads to create volume conduction (Rutkove, 2007). However, we observed no such change in CSD power. Therefore, the change in delta does appear to be due, at least in part, to the increase in goal conflict between the conditions. Changes in delta coherence during goal conflict are not present in predictions of BIS activity (Gray & McNaughton, 2000; Moore et al., 2006) or in theories of IPC (e.g., Cavanagh & Frank, 2014; Cohen, 2014). However, they are not unprecedented. For example, Papenberg, Hämmerer, Müller, Lindenberger, and Li (2013) identified increased inter-trial delta phase coherence during a go/no-go task and attributed it to increased performance monitoring. Additionally, delta coherence has been related to an increase in communications travelling from the cortex to the hippocampus during memory formation (Chan et al., 2017; Mitra et al., 2016). Therefore, delta coherence may relate to increased cortical-hippocampal communication during goal conflict. However, this is an entirely post hoc explanation and examining such a claim is beyond the scope of the present study.

### 4.3 Theta conflict activity predicts neuroticism

The regression analyses identified that activity from the high goal-conflict condition significantly predicted trait neuroticism but not trait anxiety, BIS or rBIS. Researchers have predicted that participants with greater sensitivity to threat, such as those high in neuroticism, should show heightened cortical activation during conflict (here, defined in the most general of senses) (Gray & McNaughton, 2000; Johnson, Turner, & Iwata, 2003; Neo et al., 2011). However, our results only partially align with this prediction. Specifically, three conflict theta components were included as predictor variables for neuroticism, but only the first two were positively associated with neuroticism. Furthermore, the only significant component in the model, component 3, was negatively associated with neuroticism. Additionally, trait anxiety is also used as a measure of threat sensitivity but was not significantly predicted by our model of conflict theta. Therefore, we can only offer limited support for the findings and theories of Gray and McNaughton (2000), Johnson, Turner, and Iwata (2003) and Neo et al. (2011) in terms of personality and conflict (here, goal conflict) EEG.

Contrary to predictions, theta EEG during goal conflict did not predict trait BIS or rBIS scores. We expected goal-conflict theta to predict trait BIS/rBIS for two reasons. Firstly, trait rBIS is, in theory, concerned with goal-conflict sensitivity (Torrubia, Avila, & Caseras, 2008). Secondly, previous studies have successfully linked both event related potentials (Amodio, Master, Yee, & Taylor, 2008; Balconi & Crivelli, 2010; Boksem, Tops, Wester, Meijman, & Lorist, 2006; De Pascalis, Varriale, & D’Antuono, 2010; Wacker, Chavanon, Leue, & Stemmler, 2010) and theta power (Balconi & Crivelli, 2010; Massar, Rossi, Schutter, & Kenemans, 2012; Moore et al., 2012) to trait BIS. Therefore, we expected both trait BIS and trait rBIS to be predicted by theta EEG during goal conflict. However, our rejection of this hypothesis is in line with previous applications of the calibrated SST (Neo et al., 2011; Shadli et al., 2015, 2016) that were also unable to link goal-conflict EEG to trait BIS. Concerning future investigations, Moore et al. (2012) have suggested that links between EEG and personality are most likely to be identified on the sub-second level (i.e., 100 ms or less) (Moore et al., 2012). Therefore, it may be beneficial to use EEG measures with high temporal precision that maintain spectral information, such as time frequency analysis.

### 4.4 Limitations

Finally, we should highlight three limitations of our approach that future researchers may wish to advance. Firstly, statistical research has suggested that a sample size of slightly more than 3 times of our own would be needed to produce stable regression estimates for our models (Schönbrodt & Perugini, 2013). However, this is a widespread problem that comes with the difficulties of collecting large samples within EEG research. Therefore, while our findings still contribute to the literature and have utility when compared across studies, future investigations would be better placed to consider larger sample sizes, where possible. Secondly, future research may consider splitting males and females, as both sexes appear to display unique EEG-personality links with EPQ-r traits. Specifically, research has demonstrated that neuroticism is related to a different pattern of spectral EEG in men and women, respectively (Jaušovec & Jaušovec, 2007; Razumnikova, 2004). Therefore, examining each gender individually may identify stronger links between goal-conflict-related EEG and psychometric EPQ-r traits.

Finally, the increases in theta CSD power and coherence, observed here, do align with the predictions of BIS-driven activity. Specifically, the theta waveband is thought to synchronise the structures of the BIS during goal conflict (Gray & McNaughton, 2000). However, it cannot be taken as a certainty that the coherence increases are BIS-driven, simply because they occur predominantly within the theta waveband. Therefore, future research should examine whether the goal-conflict-related increases in theta coherence observed here are reduced by anxiolytic drugs (which reduce septohippocampal-driven theta) (Gray & McNaughton, 2000; Mitchell, McNaughton, Flanagan, & Kirk, 2008). We recommend restricting the first of such studies to the MF–MPO and LF–RFC region pairs, based on their larger effect sizes, before moving onto the wider region pairs. Alternatively, studies may consider trends in theta coherence given the large number of region pairs linked to conflict in the present study. For example, researchers might examine the number of region pairs that show increases in theta during goal conflict before and after the anxiolytic drugs are administered. Furthermore, research has demonstrated that the effects of anxiolytic drugs on scalp-wide EEG can be moderated by psychometric personality scores (Perkins et al., 2013). In the present study, conflict EEG significantly predicted the psychometrically measured personality trait of neuroticism. Therefore, it is possible that neuroticism may moderate the effects of anxiolytic drugs on conflict-related theta coherence. Either way, future research should examine whether any such link between the anxiolytic drugs and the present goal-conflict-related theta effects exists.

## 5 Conclusions

In the present study, we combined the comprehensive EEG recording methodology of Moore et al. (2012) with Shadli et al.’s (2015) highly efficient calibrated SST task. In doing so, we aimed to advance EEG research into goal conflict and to provide comparisons with previous IPC research. Our results suggest that goal-conflict EEG can be understood through a network of increased theta CSD power within the MFC region, theta coherence across the scalp and delta coherence within the frontal regions. As these effects are theta-dominated and are increases in activity during goal conflict, they are aligned with the predictions of BIS-driven activity. However, future research with anxiolytic drugs is needed to confirm that these effects are BIS-driven. Compared to previous studies, our CSD power results suggest that goal-conflict EEG is characterised by a narrower spatial and frequency profile than previously thought (McNaughton et al., 2013; Moore et al., 2006, 2012; Neo et al., 2011; Shadli et al., 2015, 2016). However, our coherence results do agree with previous work demonstrating that goal conflict occurs as a scalp-wide network of theta-dominated activity (Moore et al., 2006, 2012). In comparison to IPC, our results suggest that goal conflict involves much the same profile of MFC CSD theta power activity, but that goal conflict involves a much wider network of theta coherence. Finally, our findings identified preliminary links between neuroticism and goal-conflict EEG. Overall, we here present the most comprehensive EEG analysis of Shadli et al.’s (2015) calibrated SST and, in doing so, have identified a novel network of EEG activity that is related to goal conflict and may even be BIS-driven.

## Footnotes

1 For an open-source tutorial on using CSD transforms, see Kayser and Tenke (2015).

2 The reader may wish to ask why we initially applied a common average reference, knowing that we would later transform the data into *reference-free* surface Laplacian estimates using a CSD transform. The reason for this is simply that the CSD transform must be applied to cleaned EEG data. Therefore, we needed to apply the common average reference to the data for the cleaning phase, before applying the CSD.

3 Conventionally, EEG (μV) transformed into power via an FFT uses the unit measurement μV^2^. However, CSD EEG (μV/m^2^) transformed into power via an FFT uses the unit measurement (μV/m^2^)^2^. We acknowledge this difference in unit measurement by referring to CSD EEG transformed via a FFT as CSD power. CSD power is largely comparable to conventional power (i.e., μV^2^) and has become an increasingly useful metric in the EEG literature. Specifically, CSD power acts as a measure of power which is less influenced by spatially broad effects (which are the EEG effects that are most likely to be distorted by volume conductance) (Tenke & Kayser, 2015; Tenke et al., 2017).

4 The epochs derived from the go trials would also have contained EEG activity linked to motor response superimposed onto the comparison EEG in the same epoch, but this would only have been the case in a proportion of the stop trials. However, the rate of stop trials affected in this way was very similar in the high goal-conflict and low goal-conflict conditions meaning the degree to which this would have contributed to error or artefact within the EEG DVs derived using this subtraction approach was controlled (see Section 3.1 which provides overall response trial percentages for the two experimental conditions, *low goal conflict* and *high goal conflict*).

5 It is important to point out that there was no expectation that the trials comprising the low goal-conflict condition would be completely free of goal-conflict effects, only that there would be a comparatively *lower* level of goal conflict than the trials comprising the high goal-conflict condition.

6 For the purpose of the EEG, data associated with the short SSD and long SSD stop trials were collapsed together to form the low goal-conflict condition (see Section 2.4.1) as each was reasoned to create a lower level of goal conflict than the intermediate SSD trials. However, to prove, rather than simply assume, the differences in induced goal-conflict levels, we separated the three trial types in the behavioural analyses.

7 The CSD power values and coherence values reported in the Results section are the natural log and Fisher-*Z* transformed values, respectively.

## References

[ref1] Abdi, H. (2010). The greenhouse-geisser correction In N. Salkind (Ed.), Encyclopedia of research design (pp. 1–10). Thousand Oaks, CA: Sage 10.1007/BF02289823.

[ref2] Amodio, D. M. , Master, S. L. , Yee, C. M. , & Taylor, S. E. (2008). Neurocognitive components of the behavioral inhibition and activation systems: Implications for theories of self-regulation. Psychophysiology, 45, 11–19. 10.1111/j.1469-8986.2007.00609.x. 17910730

[ref3] Andersen, S. B. , Moore, R. A. , Venables, L. , & Corr, P. J. (2009). Electrophysiological correlates of anxious rumination. International Journal of Psychophysiology, 71, 156–169. 10.1016/j.ijpsycho.2008.09.004. 18848849

[ref4] Asada, H. , Fukuda, Y. , Tsunoda, S. , Yamaguchi, M. , & Tonoike, M. (1999). Frontal midline theta rhythms reflect alternative activation of prefrontal cortex and anterior cingulate cortex in humans. Neuroscience Letters, 274, 29–32. 10.1016/S0304-3940(99)00679-5. 10530512

[ref5] Balconi, M. , & Crivelli, D. (2010). Veridical and false feedback sensitivity and punishment-reward system (BIS/BAS): ERP amplitude and theta frequency band analysis. Clinical Neurophysiology, 121, 1502–1510. 10.1016/j.clinph.2010.03.015. 20381417

[ref6] Benjamini, Y. , & Hochberg, Y. (1995). Controlling the false discovery rate: A practical and powerful approach to multiple testing. Journal of the Royal Statistical Society: Series B (Methodological), 57, 289–300. 10.1111/j.2517-6161.1995.tb02031.x.

[ref7] Bieling, P. J. , Antony, M. M. , & Swinson, R. P. (1998). The state-trait anxiety inventory, trait version: Structure and content re-examined. Behaviour Research and Therapy, 36, 777–788. 10.1016/S0005-7967(98)00023-0. 9682533

[ref8] Blume, C. , Lechinger, J. , del Giudice, R. , Wislowska, M. , Heib, D. P. J. , & Schabus, M. (2015). EEG oscillations reflect the complexity of social interactions in a non-verbal social cognition task using animated triangles. Neuropsychologia, 75, 330–340. 10.1016/j.neuropsychologia.2015.06.009. 26111488

[ref9] Boksem, M. A. S. , Tops, M. , Wester, A. E. , Meijman, T. F. , & Lorist, M. M. (2006). Error-related ERP components and individual differences in punishment and reward sensitivity. Brain Research, 1101, 92–101. 10.1016/j.brainres.2006.05.004. 16784728

[ref10] Bosch, V. , Mecklinger, A. , & Friederici, A. D. (2001). Slow cortical potentials during retention of object, spatial, and verbal information. Cognitive Brain Research, 10, 219–237. 10.1016/S0926-6410(00)00040-9. 11167047

[ref11] Botvinick, M. M. (2007). Conflict monitoring and decision making: Reconciling two perspectives on anterior cingulate function. Cognitive, Affective, & Behavioral Neuroscience, 7, 356–366. 10.3758/cabn.7.4.356. 18189009

[ref12] Botvinick, M. M. , Braver, T. S. , Barch, D. M. , Carter, C. S. , & Cohen, J. D. (2001). Conflict monitoring and cognitive control. Psychological Review, 108, 624–652. 10.1037/0033-295X.108.3.624. 11488380

[ref13] Botvinick, M. M. , Cohen, J. D. , & Carter, C. S. (2004). Conflict monitoring and anterior cingulate cortex: An update. Trends in Cognitive Sciences, 8, 539–546. 10.1016/j.tics.2004.10.003. 15556023

[ref14] Brier, M. R. , Ferree, T. C. , Maguire, M. J. , Moore, P. , Spence, J. , Tillman, G. D. , … Kraut, M. A. (2010). Frontal theta and alpha power and coherence changes are modulated by semantic complexity in Go/NoGo tasks. International Journal of Psychophysiology, 78, 215–224. 10.1016/j.ijpsycho.2010.07.011. 20696190

[ref15] Brunner, C. , Billinger, M. , Seeber, M. , Mullen, T. R. , & Makeig, S. (2016). Volume conduction influences scalp-based connectivity estimates. Frontiers in Computational Neuroscience, 10, 1–4. 10.3389/fncom.2016.00121. 27920674 PMC5119053

[ref16] Carver, C. S. , & White, T. L. (1994). Behavioral inhibition, behavioral activation, and affective responses to impending reward and punishment: The BIS/BAS scales. Journal of Personality and Social Psychology, 67, 319 10.1037/0022-3514.67.2.319.

[ref19] Cavanagh, J. F. , & Frank, M. J. (2014). Frontal theta as a mechanism for cognitive control. Trends in Cognitive Sciences, 18, 414–421. 10.1016/j.tics.2014.04.012. 24835663 PMC4112145

[ref20] Cavanagh, J. F. , & Shackman, A. J. (2015). Frontal midline theta reflects anxiety and cognitive control: Meta-analytic evidence. Journal of Physiology Paris, 109, 3–15. 10.1016/j.jphysparis.2014.04.003. PMC4213310 24787485

[ref21] Chan, R. W. , Leong, A. T. L. , Ho, L. C. , Gao, P. P. , Wong, E. C. , Dong, C. M. , … Wu, E. X. (2017). Low-frequency hippocampal–cortical activity drives brain-wide resting-state functional MRI connectivity. Proceedings of the National Academy of Sciences of the United States of America, 114, 6972–6981. 10.1073/pnas.1703309114. 28760982 PMC5565425

[ref22] Cohen, M. X. (2014). A neural microcircuit for cognitive conflict detection and signaling. Trends in Neurosciences, 37, 480–490. 10.1016/j.tins.2014.06.004. 25034536

[ref23] Cohen, M. X. , & Cavanagh, J. F. (2011). Single-trial regression elucidates the role of prefrontal theta oscillations in response conflict. Frontiers in Psychology, 2, 1–12. 10.3389/fpsyg.2011.00030. 21713190 PMC3111011

[ref110] Corr, P. J. (2008). *The reinforcement sensitivity theory of personality* Cambridge: Cambridge University Press.

[ref29] Costello, A. B. , & Osborne, J. W. (2005). Best practices in exploratory factor analysis In J. Osborne (Ed.), Best practices in quantitative methods (pp. 1–10). Thousand Oaks, CA: Sage 10.4135/9781412995627.d8.

[ref109] De Pascalis, V. , Varriale, V. , & D'Antuono, L. (2010). Event-related components of the punishment and reward sensitivity. Clinical Neurophysiology, 121, 60–76. 10.1016/j.clinph.2009.10.004. 19900840

[ref30] Dien, J. , Beal, D. J. , & Berg, P. (2005). Optimizing principal components analysis of event‐related potentials: Matrix type, factor loading weighting, extraction, and rotations. Clinical Neurophysiology, 116, 1808–1825. 10.1016/j.clinph.2004.11.025. 15996897

[ref31] Duffy, F. H. , & Als, H. (2012). A stable pattern of EEG spectral coherence distinguishes children with autism from neuro‐typical controls ‐ a large case control study. BMC Medicine, 10, 64 10.1186/1741-7015-10-64. 22730909 PMC3391175

[ref32] Eysenck, S. B. G. , Eysenck, H. J. , & Barrett, P. (1985). A revised version of the psychoticism scale. Personality and Individual Differences, 6, 21–29. 10.1016/0191-8869(85)90026.

[ref33] Ferree, T. C. , Brier, M. R. , Hart, J. , & Kraut, M. A. (2009). Space-time-frequency analysis of EEG data using within-subject statistical tests followed by sequential PCA. NeuroImage, 45, 109–121. 10.1016/j.neuroimage.2008.09.020. 18992350

[ref34] Ferreira, C. S. , Marful, A. , Staudigl, T. , Bajo, T. , & Hanslmayr, S. (2014). Medial prefrontal theta oscillations track the time course of interference during selective memory retrieval. Journal of Cognitive Neuroscience, 26, 777–791. 10.1162/jocn_a_00523. 24236766

[ref35] Franklin, S. B. , Gibson, D. J. , Robertson, P. A. , Pohlmann, J. T. , & Fralish, J. S. (1995). Parallel analysis: A method for determining significant principal components. Journal of Vegetation Science, 6, 99–106. 10.2307/3236261.

[ref111] Gray, J. A. (1982). *The neuropsychology of anxiety: An enquiry into the functions of the septo-hippocampal system* Oxford: Oxford University Press.

[ref36] Gray, J. A. , & McNaughton, N. (2000). The neuropsychology of anxiety: An enquiry into the functions of septohippocampal theories (2nd ed). Oxford: Oxford University Press 10.1093/acprof:oso/9780198522713.001.0001.

[ref37] Halliday, D. M. , & Rosenberg, J. R. (2000). On the application, estimation and interpretation of coherence and pooled coherence. Journal of Neuroscience Methods, 100, 173–174. 10.1016/S0165-0270(00)00267-3. 11040381

[ref38] Hanslmayr, S. , Pastötter, B. , Bäuml, K. H. , Gruber, S. , Wimber, M. , & Klimesch, W. (2008). The electrophysiological dynamics of interference during the Stroop task. Journal of Cognitive Neuroscience, 20, 215–225. 10.1162/jocn.2008.20020. 18275330

[ref39] Heym, N. , Ferguson, E. , & Lawrence, C. (2008). An evaluation of the relationship between Gray’s revised RST and Eysenck’s PEN: Distinguishing BIS and FFFS in Carver and White’s BIS/BAS scales. Personality and Individual Differences, 45, 709–715. 10.1016/j.paid.2008.07.013.

[ref40] Ishii, R. , Shinosaki, K. , Ukai, S. , Inouye, T. , Ishihara, T. , Yoshimine, T. , … Takeda, M. (1999). Medial prefrontal cortex generates frontal midline theta rhythm. Neuroreport, 10, 675–679. 10.3389/fnhum.2014.00406. 10208529

[ref41] Jarovi, J. , Volle, J. , Yu, X. , Guan, L. , & Takehara-Nishiuchi, K. (2018). Prefrontal theta oscillations promote selective encoding of behaviorally relevant events. Eneuro, 5, pii: ENEURO.0407–18.2018. 10.1523/eneuro.0407-18.2018. PMC6348453 30693310

[ref42] Jaušovec, N. , & Jaušovec, K. (2007). Personality, gender and brain oscillations. International Journal of Psychophysiology, 66, 215–224. 10.1016/j.ijpsycho.2007.07.005. 17761331

[ref43] Johnson, S. L. , Turner, R. J. , & Iwata, N. (2003). BIS/BAS levels and psychiatric disorder: An epidemiological study. Journal of Psychopathology and Behavioral Assessment, 25, 25–36. 10.1023/A:1022247919288.

[ref45] Jung, T. , Makeig, S. , Humphries, C. , Lee, T. , McKeown, M. J. , Iragui, I. , & Sejnowski, T. J. (2000). Removing electroencephalographic artefacts by blind source separation. Psychophysiology, 37, 163–178. 10.1111/1469-8986.3720163. 10731767

[ref46] Kayser, J. , & Tenke, C. E. (2003). Optimizing PCA methodology for ERP component identification and measurement: Theoretical rationale and empirical evaluation. Clinical Neurophysiology, 114, 2307–2325. 10.1016/S1388-2457(03)00241-4. 14652090

[ref47] Kayser, J. , & Tenke, C. E. (2006). Principal components analysis of Laplacian waveforms as a generic method for identifying ERP generator patterns: II. Adequacy of low-density estimates. Clinical Neurophysiology, 117, 369–380. 10.1016/j.clinph.2005.08.033. 16356768

[ref48] Kayser, J. , & Tenke, C. E. (2010). In search of the Rosetta Stone for scalp EEG: converging on reference-free techniques. Clinical Neurophysiology: Official Journal of the International Federation of Clinical Neurophysiology, 121, 1973–1975. 10.1016/j.clinph.2010.04.030. 20566375 PMC2953588

[ref49] Kayser, J. , & Tenke, C. E. (2015). Issues and considerations for using the scalp surface Laplacian in EEG/ERP research: A tutorial review. International Journal of Psychophysiology, 97, 189–209. 10.1016/j.ijpsycho.2015.04.012. 25920962 PMC4537804

[ref50] Khadem, A. , & Hossein-Zadeh, G. A. (2014). Quantification of the effects of volume conduction on the EEG/MEG connectivity estimates: An index of sensitivity to brain interactions. Physiological Measurement, 35, 2149–2164. 10.1088/0967-3334/35/10/2149. 25243864

[ref52] Lachaux, J.-P. , Rodriguez, E. , Martinerie, J. , & Varela, F. J. (1999). Measuring phase synchrony in brain signals. Human Brain Mapping, 8, 194–208. 10.1002/(SICI)1097-0193(1999)8:4. 10619414 PMC6873296

[ref55] Luck, S. J. , & Gaspelin, N. (2017). How to get statistically significant effects in any ERP experiment (and why you shouldn’t). Psychophysiology, 54, 146–157. 10.1111/psyp.12639. 28000253 PMC5178877

[ref58] Massar, S. A. A. , Rossi, V. , Schutter, D. J. L. G. , & Kenemans, J. L. (2012). Baseline EEG theta/beta ratio and punishment sensitivity as biomarkers for feedback-related negativity (FRN) and risk-taking. Clinical Neurophysiology, 123, 1958–1965. 10.1016/j.clinph.2012.03.005. 22542439

[ref57] McDonald, J. H. (2008). Handbook of biological statistics (2nd ed). Baltimore, MD: Sparky House.

[ref59] McNaughton, N. , & Corr, P. J. (2004). A two-dimensional neuropsychology of defense: Fear/anxiety and defensive distance. Neuroscience and Biobehavioral Reviews, 28, 285–305. 10.1016/j.neubiorev.2004.03.005. 15225972

[ref61] McNaughton, N. , DeYoung, C. G. , & Corr, P. J. (2016). Approach/avoidance In J. R. Absher & J. Cloutier (Eds.), Neuroimaging personality, social cognition, and character (pp. 25–49). San Diego, CA: Elsevier Academic Press 10.1016/B978-0-12-800935-2.00002-6.

[ref62] McNaughton, N. , Swart, C. , Neo, P. , Bates, V. , & Glue, P. (2013). Anti-anxiety drugs reduce conflict‐specific theta ‐ a possible human anxiety-specific biomarker. Journal of Affective Disorders, 148, 104–111. 10.1016/j.jad.2012.11.057. 23261140

[ref64] Mitchell, D. J. , McNaughton, N. , Flanagan, D. , & Kirk, I. J. (2008). Frontal-midline theta from the perspective of hippocampal “theta.” Progress in Neurobiology, 86, 156–185. 10.1016/j.pneurobio.2008.09.005. 18824212

[ref65] Mitra, A. , Snyder, A. Z. , Hacker, C. D. , Pahwa, M. , Tagliazucchi, E. , Laufs, H. , … Raichle, M. E. (2016). Human cortical–hippocampal dialogue in wake and slow-wave sleep. Proceedings of the National Academy of Sciences of the United States of America, 113, 6868–6876. 10.1073/pnas.1607289113. 27791089 PMC5098641

[ref66] Moore, R. A. , Gale, A. , Morris, P. H. , & Forrester, D. (2006). Theta phase locking across the neocortex reflects cortico-hippocampal recursive communication during goal conflict resolution. International Journal of Psychophysiology, 60, 260–273. 10.1016/j.ijpsycho.2005.06.003. 16168505

[ref67] Moore, R. A. , Gale, A. , Morris, P. H. , & Forrester, D. (2008). Alpha power and coherence primarily reflect neural activity related to stages of motor response during a continuous monitoring task. International Journal of Psychophysiology, 69, 79–89. 10.1016/j.ijpsycho.2008.03.003. 18430481

[ref68] Moore, R. A. , Mills, M. , Marshman, P. , & Corr, P. J. (2012). Behavioural Inhibition System (BIS) sensitivity differentiates EEG theta responses during goal conflict in a continuous monitoring task. International Journal of Psychophysiology, 85, 135–144. 10.1016/j.ijpsycho.2012.06.006. 22732350

[ref70] Neo, P. S. H. , Thurlow, J. K. , & McNaughton, N. (2011). Stopping, goal-conflict, trait anxiety and frontal rhythmic power in the stop-signal task. Cognitive, Affective, & Behavioral Neuroscience, 11, 485–493. 10.3758/s13415-011-0046-x. 21647572

[ref71] Nigbur, R. , Cohen, M. X. , Ridderinkhof, K. R. , & Sturmer, B. (2012). Theta dynamics reveal domain-specific control over stimulus and response conflict. Journal of Cognitive Neuroscience, 24, 1264–1274. 10.1162/jocn_a_00128. 21861681

[ref72] Nigbur, R. , Ivanova, G. , & Stürmer, B. (2011). Theta power as a marker for cognitive interference. Clinical Neurophysiology, 122, 2185–2194. 10.1016/j.clinph.2011.03.030. 21550845

[ref73] Nunez, P. L. , & Srinivasan, R. (2006). Electric fields of the brain: The neurophysics of EEG. Oxford: Oxford University Press 10.1093/acprof:oso/9780195050387.001.0001.

[ref74] O'Neill, P.-K. , Gordon, J. A. , & Sigurdsson, T. (2013). Theta oscillations in the medial prefrontal cortex are modulated by spatial working memory and synchronize with the hippocampus through its ventral subregion. Journal of Neuroscience, 33, 14211–14224. 10.1523/jneurosci.2378-13.2013. 23986255 PMC3756763

[ref75] Onton, J. , Delorme, A. , & Makeig, S. (2005). Frontal midline EEG dynamics during working memory. NeuroImage, 27, 341–356. 10.1016/j.neuroimage.2005.04.014. 15927487

[ref77] Padrão, G. , Rodriguez-Herreros, B. , Pérez Zapata, L. , & Rodriguez-Fornells, A. (2015). Exogenous capture of medial-frontal oscillatory mechanisms by unattended conflicting information. Neuropsychologia, 75, 458–468. 10.1016/j.neuropsychologia.2015.07.004. 26151855

[ref78] Papenberg, G. , Hämmerer, D. , Müller, V. , Lindenberger, U. , & Li, S. C. (2013). Lower theta inter-trial phase coherence during performance monitoring is related to higher reaction time variability: A lifespan study. NeuroImage, 83, 912–920. 10.1016/j.neuroimage.2013.07.032. 23876249

[ref79] Perkins, A. M. , Ettinger, U. , Weaver, K. , Schmechtig, A. , Schrantee, A. , Morrison, P. D. , … Corr, P. J. (2013). Advancing the defensive explanation for anxiety disorders: Lorazepam effects on human defense are systematically modulated by personality and threat-type. Translational Psychiatry, 3, e246 10.1038/tp.2013.20. 23591970 PMC3641407

[ref80] Perrin, F. , Pernier, J. , Bertrand, O. , & Echallier, J. F. (1989). Spherical splines for scalp potential and current density mapping. Electroencephalography and Clinical Neurophysiology, 72, 184–187. 10.1016/0013-4694(89)90180-6. 2464490

[ref82] Pinner, J. F. L. , & Cavanagh, J. F. (2017). Frontal theta accounts for individual differences in the cost of conflict on decision making. Brain Research, 1672, 73–80. 10.1016/j.brainres.2017.07.026. 28778686 PMC5578402

[ref83] Razumnikova, O. M. (2004). Gender differences in hemispheric organization during divergent thinking: an EEG investigation in human subjects. Neuroscience Letters, 362, 193–195. 10.1016/j.neulet.2004.02.066. 15158012

[ref84] Reuter, M. , Cooper, A. J. , Smillie, L. D. , Markett, S. , & Montag, C. (2015). A new measure for the revised reinforcement sensitivity theory: Psychometric criteria and genetic validation. Frontiers in Systems Neuroscience, 9, 1–38. 10.3389/fnsys.2015.00038. 25852497 PMC4360558

[ref85] Ridderinkhof, K. R. , Ullsperger, M. , Crone, E. A. , & Nieuwenhuis, S. (2004). The role of the medial frontal cortex in cognitive control. Science, 306, 443–447. 10.1126/science.1100301. 15486290

[ref88] Rutkove, S. B. (2007). Introduction to volume conduction In A. S. Blum & S. B. Rutkove (Eds.), The clinical neurophysiology primer (pp. 43–53). Totowa, NJ: Humana Press 10.1007/978-1-59745-271-7_4.

[ref89] Salkind, N. J. (Ed.). (2010). Encyclopedia of research design. Thousand Oaks, CA: Sage 10.4135/9781412961288.

[ref90] Sarnthein, J. , Petsche, H. , Rappelsberger, P. , Shaw, G. L. , & von Stein, A. (1998). Synchronization between prefrontal and posterior association cortex during human working memory. Proceedings of the National Academy of Sciences of the United States of America, 95, 7092–7096. 10.1073/pnas.95.12.7092. 9618544 PMC22750

[ref91] Schönbrodt, F. D. , & Perugini, M. (2013). At what sample size do correlations stabilize? Journal of Research in Personality, 47, 609–612. 10.1016/j.jrp.2013.05.009.

[ref92] Sehatpour, P. , Molholm, S. , Schwartz, T. H. , Mahoney, J. R. , Mehta, A. D. , Javitt, D. C. , … Foxe, J. J. (2008). A human intracranial study of long-range oscillatory coherence across a frontal-occipital-hippocampal brain network during visual object processing. Proceedings of the National Academy of Sciences of the United States of America, 105, 4399–4404. 10.1073/pnas.0708418105. 18334648 PMC2393806

[ref93] Shadli, S. M. , Glue, P. , McIntosh, J. , & McNaughton, N. (2015). An improved human anxiety process biomarker: Characterization of frequency band, personality and pharmacology. Translational Psychiatry, 5, e699 10.1038/tp.2015.188 26670284 PMC5068587

[ref94] Shadli, S. M. , Smith, M. J. , Glue, P. , & McNaughton, N. (2016) Testing an anxiety process biomarker: Generalisation from an auditory to a visual stimulus. Biological Psychology, 117, 50–55. 10.1016/j.biopsycho.2016.02.011. 26944203

[ref95] Spielberger, C. D. (1983). Manual for the state-trait anxiety inventory STAI. Palo Alto, CA: Consulting Psychologists Press.

[ref96] Stins, J. F. , Polderman, J. C. , Boomsma, D. I. , & de Geus, E. J. (2005). Response interference and working memory in 12-year-old children. Child Neuropsychology, 11, 191–201. 10.1080/092970490911351. 16036444

[ref98] Tenke, C. E. , & Kayser, J. (2015). Surface Laplacians (SL) and phase properties of EEG rhythms: Simulated generators in a volume-conduction model. International Journal of Psychophysiology, 97, 285–298. 10.1016/j.ijpsycho.2015.05.008. 26004020 PMC4537832

[ref99] Tenke, C. E. , Kayser, J. , Svob, C. , Miller, L. , Alvarenga, J. E. , Abraham, K. , … Bruder, G. E. (2017). Association of posterior EEG alpha with prioritization of religion or spirituality: A replication and extension at 20-year follow-up. Biological Psychology, 124, 79–86. 10.1016/j.pain.2014.05.025. 28119066 PMC5329132

[ref100] Torrubia, R. , Avila, C. , & Caseras, X. (2008). Reinforcement sensitivity scales In P. J. Corr (Ed.), The reinforcement sensitivity theory of personality (pp. 188–227). Cambridge: Cambridge University Press 10.1017/CBO9780511819384.007.

[ref102] van de Vijver, I. , Ridderinkhof, K. R. , & Cohen, M. X. (2011). Frontal oscillatory dynamics predict feedback learning and action adjustment. Journal of Cognitive Neuroscience, 23, 4106–4121. 10.1162/jocn_a_00110. 21812570

[ref101] van den Broek, S. P. , Reinders, F. , Donderwinkel, M. , & Peters, M. J. (1998). Volume conduction effects in EEG and MEG. Electroencephalography and Clinical Neurophysiology, 106, 522–534. 10.1016/S0013-4694(97)00147-8. 9741752

[ref104] Wacker, J. , Chavanon, M. L. , Leue, A. , & Stemmler, G. (2010). Trait BIS predicts alpha asymmetry and P300 in a go/no-go task. European Journal of Personality, 24, 85–105. 10.1002/per.740

[ref105] Young, C. K. , & McNaughton, N. (2009). Coupling of theta oscillations between anterior and posterior midline cortex and with the hippocampus in freely behaving rats. Cerebral Cortex, 19, 24–40. 10.1093/cercor/bhn055. 18453538

[ref108] Zavala, B. , Tan, H. , Ashkan, K. , Foltynie, T. , Limousin, P. , Zrinzo, L. , … Brown, P. (2016). Human subthalamic nucleus-medial frontal cortex theta phase coherence is involved in conflict and error related cortical monitoring. NeuroImage, 137, 178–187. 10.1016/j.neuroimage.2016.05.031. 27181763 PMC4927260

